# Thinking in Terms of Structure-Activity-Relationships (T-SAR): A Tool to Better Understand Nanofiltration Membranes

**DOI:** 10.3390/membranes1030162

**Published:** 2011-07-15

**Authors:** José F. Fernández, Bernd Jastorff, Reinhold Störmann, Stefan Stolte, Jorg Thöming

**Affiliations:** 1 UFT, Department of Chemical Engineering—Recovery and Recycling, University of Bremen, Bremen, 28359, Germany; E-Mail: thoeming@uni-bremen.de; 2 UFT, Department of Sustainable Chemistry, University of Bremen, Bremen, 28359, Germany; E-Mails: bernd.jastorff@t-online.de (B.J.); rstoerm@uni-bremen.de (R.S.); sstolte@uni-bremen.de (S.S.); 3 CHEOPS Dienstleistungs-GmbH, Bremen, 28217, Germany

**Keywords:** nanofiltration, structure-activity-relationships, ionic liquid, stereochemistry, molecular interaction potential, reactivity, property, performance, characterization method

## Abstract

A frontier to be conquered in the field of membrane technology is related to the very limited scientific base for the rational and task-specific design of membranes. This is especially true for nanofiltration membranes with properties that are based on several solute-membrane interaction mechanisms. “Thinking in terms of Structure-Activity-Relationships” (T-SAR) is a methodology which applies a systematic analysis of a chemical entity based on its structural formula. However, the analysis become more complex with increasing size of the molecules considered. In this study, T-SAR was combined with classical membrane characterization methods, resulting in a new methodology which allowed us not only to explain membrane characteristics, but also provides evidence for the importance of the chemical structure for separation performance. We demonstrate an application of the combined approach and its potential to discover stereochemistry, molecular interaction potentials, and reactivity of two FilmTec nanofiltration membranes (NF-90 and NF-270). Based on these results, it was possible to predict both properties and performance in the recovery of hydrophobic ionic liquids from aqueous solution.

## 1. Introduction

Ionic liquids are compounds that consist exclusively of ions and have melting points below 100 °C [1]. They are regarded as a promising substance class; because they are potential substituents for volatile solvents as well as allowing the design of new processes. However, until now, only a few industrial processes have used them [2]. One of the reasons is related to handling wastes containing the contamination of possibly toxic and not easily biodegradable ionic liquids. As a direct consequence, an application of ionic liquids should minimize waste generation and allow the recovery of the ionic liquids, especially from wastewaters [3].

Nanofiltration seems to be a versatile method for this task, because nanofiltration membranes can be selected properly according to the required purpose: either to retain the ionic liquid or to allow it to pass through the membrane [4]. However, in the field of membrane technology, the scientific base for the rational and task-specific design of membranes is very limited. This is especially true for nanofiltration membranes, the performance of which is based on several solute-membrane interaction mechanisms. Therefore, any deeper understanding of these mechanisms will improve the predictability of the separation behavior of nanofiltration membranes.

**Figure 1:**
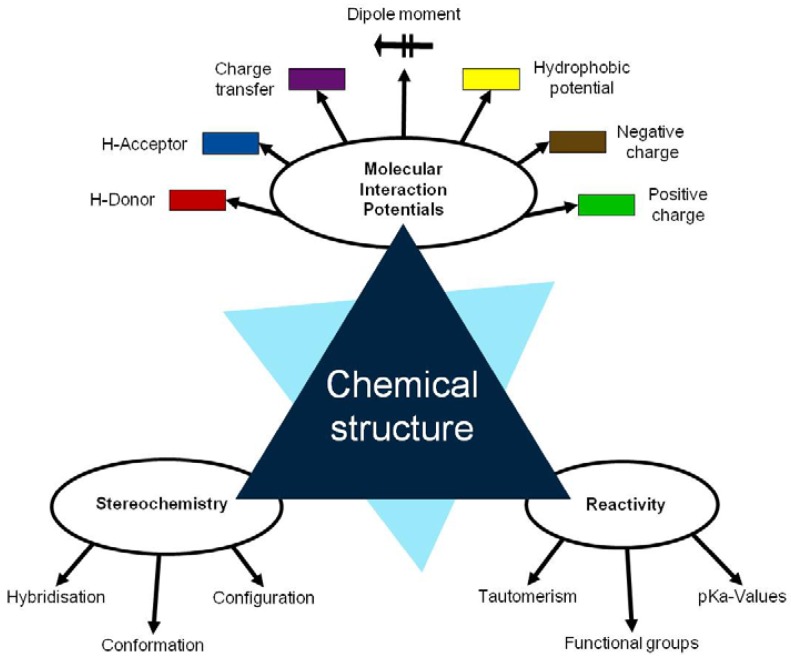
The “Thinking in terms of Structure-Activity-Relationships” (T-SAR) triangle (Adapted from [6]).

The fundamentals of the methodology denominated in “Thinking in terms of Structure-Activity Relationships” (T-SAR) were published by Jastorff, Störmann and Wölcke in 2003 [5]. T-SAR applies a systematic analysis of a chemical entity based on its structural formula and allows the formulation of working hypotheses about properties and effects of chemicals that have not yet been experimentally verified [6]. Indeed, it has been applied with success to determine the properties and the effects of different substance classes on biological systems, like ionic liquids [7,8,9], biocides [10,11] and chitosan [12]. The T-SAR approach can be represented as a triangle, as in Figure 1.

The chemical structure should ideally be stated in its corresponding three-dimensional formula, which is the graphical representation of the molecular structure, showing how the atoms are arranged. From this structure it is necessary to systematically identify several aspects related to the stereochemistry, the molecular interaction potentials and the reactivity of the desired compound; these three parameters constitute the cornerstones of the T-SAR analysis.

The stereochemistry describes the shape of the molecule on the one hand and its flexibility on the other. Thus it also describes directionality and spatial organization of molecular interaction potentials, which are identified by using a color code (Figure 1). These interaction potentials describe the possible attractions and repulsions within a molecule, or between different molecules or molecular superstructures. Finally, the reactivity indicates the potential of the molecule for further transformation and it is related with the presence of functional groups, tautomerism and pKa-values [5,6]. A practically oriented algorithm for the T-SAR approach implies the observance of seventeen steps, organized in Table 1.

**Table 1:** Algorithm for the T-SAR analysis of a chemical compound.

**Chemical structure**	Identify the atoms from the structural formula Identify the types of bond that are present Localize free electron pairs
**Stereochemistry**	4 Identify the hybridization of all the atoms 5 Identify ring systems and their stereochemical features 6 Identify steric hindrance and conformational freedom 7 Identify possible geometric isomerism 8 Determine the presence of chiral centers
**Molecular Interaction Potentials**	9 Identify hydrogen bond donor potential 10 Identify hydrogen bond acceptor potential 11 Identify charge transfer (π -π) interaction potential 12 Identify groups with local dipole moments 13 Identify groups with hydrophobic interaction potential 14 Identify permanently charged groups (ionic potential)
**Reactivity**	15 Identify possibilities for prototropic shifts (tautomerism) 16 Estimate pKa values for groups able to accept or donate protons 17 Identify remaining functional groups and their reactivity

By following these steps it is possible to analyze every compound using only its three-dimensional chemical structure, but the analysis becomes more complex as the size of the molecule increases. According to this, the following key questions were propounded for this study:

(1) Considering that the interactions between the chemical structures of the ionic liquid and the membrane are responsible for the separation, is the T-SAR methodology able to provide a better picture of a nanofiltration membrane?
(2) Can such a model be used to understand membrane properties and also predict the performance of nanofiltration membranes for the recovery of ionic liquids? Are these predictions in agreement with experimental data?

## 2. Experimental Section

Two FilmTec nanofiltration membranes (NF-90 and NF-270) were obtained from the Dow Chemical Company (Midland, USA), while three ionic liquids containing the bis(trifluoromethyl-sulfonyl)amide anion, (CF3SO2)2N, were obtained from Merck KGaA (Darmstadt, Germany). The following cations were used: 1-hexyl-3-methylimidazolium (IM16), 1-hexylpyridinium (Py6) and 1-hexyl-1-methylpyrrolidinium (Pyr16). To reduce the influence of additional ions, deionised water was used to prepare model solutions of ionic liquids.

Attenuated Total Reflection (ATR) Fourier-Transform Infrared (FTIR) spectroscopy was used to confirm the chemical composition of membrane samples. ATR-FTIR spectra were obtained using a Thermo Nicolet Avatar 370 FTIR spectrometer (Thermo Electron Corporation) equipped with an ATR element (zinc selenide crystal) and the Omnic software. An instrument blank was taken to account for the differences in instrument response and atmospheric environment. The membrane active layers were pressed tightly against the crystal plate. At least four replicates were obtained for every membrane, with each spectrum being averaged from 200 scans collected from 650 to 4000 cm^−1^ at 4 cm^−1^ resolution, and rationed to the appropriate background spectra. No baseline or further ATR corrections were applied. The absorbance intensities were normalized for further comparison between the spectra.

The streaming potential measurements were performed by Anton Paar GmbH (Graz, Austria) with the SurPASS Adjustable Gap Cell in presence of a 5 mM solution of KCl as background electrolyte at different pH values, which was adjusted with 0.1 M HCl or 0.1 M NaOH solutions. For each measurement, a pair of membrane pieces (cross section of 20 × 10 mm^2^) was used. The electrolyte pH was first decreased by adding 0.1 M HCl. After that, the same membrane samples were rinsed with deionised water and fresh 5 mM KCl solution and titration continues towards the alkaline range using 0.1 M NaOH.

For the determination of pure water permeability, the membranes were placed in deionised water for two days before use to assure complete swelling. For the experiments, a stirred dead-end cell HP4750 (Sterlitech Corporation) with a membrane active area of 13.9 cm^2^ was used. The feed pressure was achieved by an inert nitrogen atmosphere and experiments were carried out at ambient temperature. The swollen membrane was conditioned with deionised water by increasing pressure progressively in 10 bar steps until a final pressure of 40 bar was reached. After that, deionised water was filled in the cell again; operation pressure difference was fixed (from 10 to 40 bar, increasing the pressure by 5 bar every time) and the time required to collect 25 mL permeate volume was measured. Values reported are the averages of at least five measurements using a new membrane piece in each case.

For the experiments using ionic liquids, the same stirred dead-end cell was used. Saturated feed solutions were prepared by using an excess of ionic liquid, according to the water solubility values already known [13]. Swollen membranes were conditioned as described above. After that, 100 mL of feed at 25 °C were filled into the cell, the pressure difference was fixed at 35 bar and 70 mL permeate were removed. The time required to obtain this volume was measured. Samples of feed, retentate and cumulated permeate were taken for analysis by ion-chromatography (IC).

Such measurements were carried out using a Metrohm 881 Compact IC system with Metrohm accessories and software (Herisau, Switzerland). It is equipped with an online eluent degasser, a 20 μL injection loop and a conductometric detector maintained at 30 °C. All chromatographic data were recorded by the MagICNet 1.1 compact software.

For cation determinations, a C4 ion exchange column (50 × 4.0 mm ID and 5 μm mean particle size) coupled with C4 Guard and RP Guard was used. A flow rate of 0.9 mL/min eluent (2 mM HNO_3_, 30% CH_3_CN) was applied. For the determination of anions an A Supp ion exchange column (50 × 4.0 mm ID and 5 μm mean particle size) coupled with A Supp 4/5 Guard and RP Guard was used. A flow rate of 0.7 mL/min eluent (3.2 mM Na_2_CO_3_, 2 mM NaHCO_3_, 35% CH_3_CN) was applied. Additionally, a self-regenerating Suppressor Module and a CO_2_-Suppressor were used.

## 3. Results and Discussion

### 3.1. T-SAR Algorithm Applied to Nanofiltration Membranes

The separation process in nanofiltration membranes is mainly controlled by the chemistry of the ultrathin barrier layer, by phenomena taking place either at the surface or the bulk of this layer. Consequently, the main task is related to following the seventeen steps of the T-SAR algorithm for only the top layer of each membrane.

Dow produces two different types of FilmTec polyamide membranes based on the early membranes developed and patented by Cadotte [14,15,16]: the first type is a fully aromatic polyamide and the second type is a mixed aromatic-aliphatic polyamide. Both types of membranes are produced from the reaction of aromatic trimesoyl chloride (benzene-1,3,5-tricarbonyl chloride) with the corresponding diamine: m-phenylene diamine (aromatic) or piperazine (aliphatic). Their chemical structures are represented in Table 2.

**Table 2:** Monomers involved in the chemistry of FilmTec nanofiltration (NF)-membranes and their pKa values.

**Compound**	**Chemical structure**	**pKa values [17]**
Trimesoyl chloride	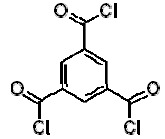	3.12, 3.89, 4.70 (Trimesic acid)
m-phenylene diamine	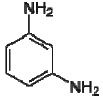	4.88, 2.65
Piperazine	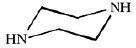	9.82, 5.68

**Figure 2:**
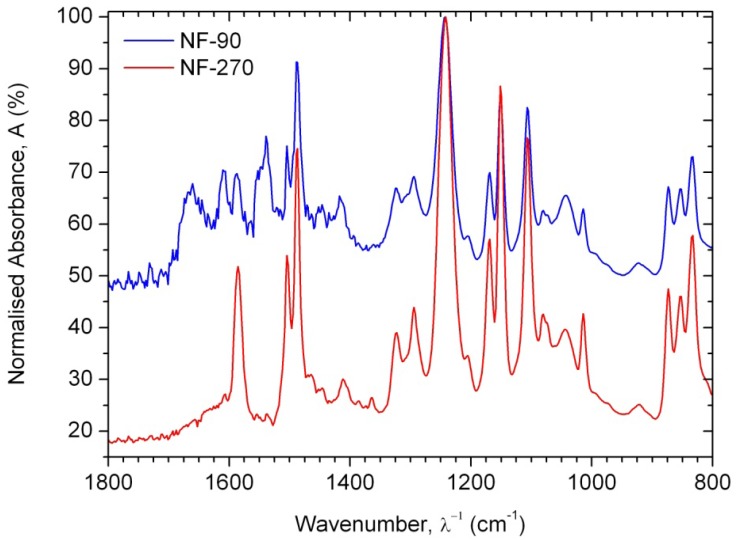
ATR-FTIR spectra for nanofiltration membranes over 800–1800 cm^−1^.

As consequence, the differences observed in the ATR-FTIR spectra (Figure 2) are due to the polyamide formed during the interfacial polymerization reaction, while the similarities are due to the presence of a common polysulfone layer acting as microporous substrate. Due to the presence of the characteristic Amide I (1680–1630 cm^−1^) and Amide II (1570–1515 cm^−1^) peaks, it is possible to confirm that the NF-90 membrane is an aromatic polyamide, while the NF-270 membrane is therefore a mixed aliphatic-aromatic polyamide. It represents a clear advantage for the T-SAR analysis: trimesoyl chloride takes part in both structures, meaning that the differences between both membranes in terms of properties and performance should be associated with the difunctional amine used in each case.

The analysis of the stereochemistry begins by determining the hybridization of each atom: *sp^2^* for each atom forming part of aromatic rings and amide bonds, *sp^3^* for atoms forming part of the piperazine ring and *s* for all hydrogen atoms involved. Then, two different geometries can be identified: planar aromatic rings derived from trimesoyl chloride and m-phenylene diamine, and spatial heterocyclic rings derived from piperazine. Furthermore, the trans-isomer (or Z-configuration) is expected to be the most preferred configuration for the planar amide bonds formed between trimesoyl chloride and m-phenylene diamine due to steric factors and charge interactions [18,19]. In the case of piperazine, the chair conformation is more energetically favored by the boat conformation, when the piperazine ring is forming part of an amide bond [20,21]. Finally, there is no evidence of chiral centers, due to the absence of *sp^3^* hybridized carbon atoms with four different substituents.

Acyl chloride groups (-COCl) can react either with the amino groups or with water, therefore two possible main constituting units for both polyamide structures are possible: the linear one, in which only two of the three acyl chloride groups are forming amide bonds and the third one reacts with water to form a carboxylic acid group; and the cross-linked unit, in which every acyl chloride group forms amide bonds. Additionally, the polymeric chains could finish either with an unreacted amino group or with a carboxylic acid group, a product of the hydrolysis of the acyl chloride group.

Table 3 shows such constituting units, drawn with respect to bond angles and spatial distribution of the atoms. However, lone pairs of electrons are not represented. Furthermore, using the color code developed as part of the T-SAR algorithm (Figure 1), it is possible to obtain a map of inherent interaction potentials for the membrane constituent units under observation (see also Table 3).

**Table 3:** Stereochemistry and molecular interaction potentials of FilmTec NF-membranes constituting units, according to the color code from Figure 1.

**Constituting unit**	**Membrane**	
	**NF-90**	**NF-270**
Linear	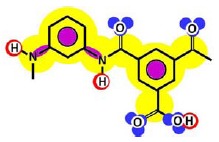	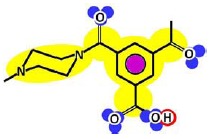
Cross-linked	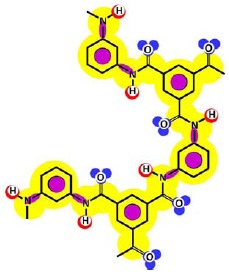	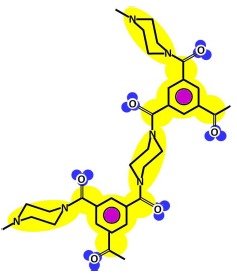
Amino end-group	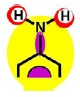	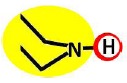
Carboxylic acid end-group	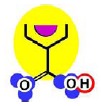	

In the case of the NF-90 membrane, strong H-donor and H-acceptor potentials are represented by red and blue colors. Strong charge transfer potentials are found in both types of aromatic rings, identified by violet color; while a permanent dipole exist in the aromatic ring derived from m-phenylene diamine, due to the 1,3-distribution of both amino groups. Contrarily, in the case of the NF-270 membrane H-donor potentials (red-colored) are limited to the end groups, while strong H-acceptor potentials (blue-colored) dominate the interaction map for this membrane. Moreover, the charge transfer interaction potential is limited only to the aromatic rings and no dipole moments can be identified. For both membranes, the skeleton formed by all carbon and nitrogen atoms exhibits hydrophobic potential and thus is colored yellow.

As it can be observed, membranes in the dry state do not possess permanently charged groups. However, both amino and carboxylic acid end groups are responsible for the electrical charge that nanofiltration membranes acquire in contact with water. In a wet state, they can accept or donate a proton respectively, changing their interaction potentials to ionic interaction potentials (colored green for amino groups and brown for carboxylic acid groups). The changes introduced by water in the interaction potential map of both membranes are represented in Table 4.

**Table 4:** Molecular interaction potentials for charged groups of FilmTec NF-membranes, according to the color code from Figure 1.

**Constituting unit**	**Membrane**	**Corresponding reaction**
Amino end-group	NF-90	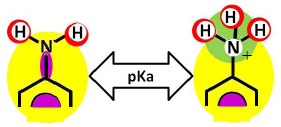
	NF-270	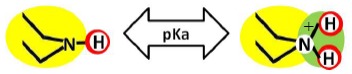
Carboxylic acid group (linear unit or end-group)	Both membranes	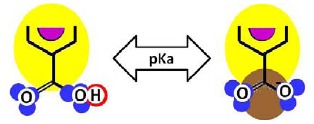

The analysis of membrane reactivity begins with the identification of possibilities for prototropic shifts, referring to the relocation of protons. In this case, the only shift possible is related to the presence of hydrogen in the amide bond of fully aromatic polyamides (NF-90), but on account of both greater bond energies and resonance energies, the amide-imidol tautomerism is not possible [22].

The identification of pKa values for the functional groups with H-acceptor or H-donor potential is not an easy task. The pKa values derived from monomers (Table 2) must be considered only as a first approximation to the real ones, which are conditioned by the ring substitutions and/or the polymerization effects. If chemical compounds similar to some parts of the membrane structures exist, then their pKa values could be used as a refined approximation.

Finally, as the last step of the T-SAR algorithm indicates, it is necessary to identify other kinds of reactivity for each functional group involved. From all possible reactions which could take place from the chemical point of view, the electrophilic substitution for the aromatic ring, the hydrolysis of the amide bond, and the oxidation and salt formation for both end groups; have a certain probability of occurring in aqueous applications of such membranes.

### 3.2. A Better Picture of both Nanofiltration Membranes

It is supposed that the membrane begins to grow in the plane of polymerization, corresponding to the interface between the organic phase containing trimesoyl chloride and the aqueous solution containing the diamine. After that, additional diamine monomers which are able to diffuse through the first formed polymer monolayer begin the process again in a subsequent membrane monolayer. All the monolayers which constitute the top layer are kept together either by cross-linking reactions or by attractions derived from molecular interaction potentials.

In this context, the chemical structure of each membrane monolayer is a consequence of a given combination between linear and cross-linked constituting units, as well as both kinds of end-groups. At this point, experimental information derived from X-ray photoelectron spectroscopy (XPS) analysis can be helpful to reveal how such combination of constituting units looks for the upper monolayer of the top layer. The ratio oxygen-nitrogen (O/N) is a measure for the membrane cross-linking degree [23]. Considering that each membrane monolayer comprises “m” linear units and “n” cross-linked units, it is possible to determine the ratio of cross-linked to linear units, as follows:

(1) $$\overset{\text{n}}{\;}/\underset{\text{m}}{\;}=\frac{2-O/N}{3\cdot (O/N-1)}$$

where: n/m: Cross-linked to linear units ratio (−); O/N = oxygen to nitrogen ratio (−).

An O/N ratio of 2 represents a fully linear polyamide structure (n = 0), while an O/N ratio of 1 represents a fully cross-linked polyamide structure (m = 0). Using XPS experimental data recently published for both FilmTec membranes [24], the n/m ratio for the NF-90 membrane can be represented as a fractional number by 9/5 (O/N = 1.16 ± 0.16). In a similar way, an n/m ratio for the NF-270 membrane can be also obtained and it can be represented as a fractional number by 3/4 (O/N = 1.31 ± 0.09). However, an equivalent value of 6/8 was selected for this study to assure that the total number of constituting units is the same in both cases (14 in total).

According to Table 5, the number of linear and cross-linked constituting units defines how many trifunctional and difunctional monomers are involved. Trimesoyl chloride can build three covalent bonds; while both amines can build only two covalent bonds. As a consequence, some covalent bonds remain “available” for further growth of the polymeric structure.

**Table 5:** Basic information needed to assemble the membrane patterns.

**Number of**	**Membrane**	

	**NF-90**	**NF-270**
**Linear units (m)**	5	8
**Cross-linked units (n)**	9	6
**Trifunctional monomers**	23	20
**Difunctional monomers**	32	26
**Remaining bonds**	5	8

With all these assumptions in mind, the constituting units should be randomly arranged to form the polyamide structure, but it should be coherent with the properties exhibited by the membrane and able to describe them in an adequate way. Because several arrangements may differ in the number and nature of end-groups, the determination of the isoelectric point (IEP) in the wet state can be a useful way to check such coherence, considering that the IEP is the pH at which a particular molecule carries no electrical charge. In the case of nanofiltration membranes, it can be calculated as follows:

(2) $$-\sum {\text{N}}_{{\text{COO}}^{-}}\cdot {\left(\frac{\%{\text{COO}}^{-}}{100}\right)}_{\text{pH}=\text{IEP}}+{\text{N}}_{{\text{NH}}_{\text{X}}^{+}}\cdot {\left(\frac{\%{\text{NH}}_{\text{X}}^{+}}{100}\right)}_{\text{pH}=\text{IEP}}=0$$

where:

N_COO_^−^: number of deprotonated carboxylic acid groups (−);
%COO^−^: amount of deprotonated carboxylic acid groups already formed at pH = IEP (%);
N_NHx_^+^: number of protonated amino groups (−);
%NHx^+^: amount of protonated amino groups already formed at pH = IEP (%).

The amount of the protonated form of carboxylic acid and amino groups is pH dependent and can be calculated from pKa values and acid-base dissociation equilibrium. As it was pointed out above, Table 6 summarizes the pKa values for some chemical compounds, which were calculated using MOPAC2009™ calculations tools for pKa determination in the case of carboxylic acid groups [25] or were found in the CAS-databases in the case of amino groups [26].

**Table 6:** pKa values for compounds with a chemical structure similar than that found in the membrane's chemistry.

**Type of group**	**NF-90**		**NF-270**	
	**pKa value**	**Derived from**	**pKa value**	**Derived from**
Carboxylic acid group (linear unit)	2.52	3,5-bis [(phenylamino)carbonyl]-benzoic acid	2.56	3,5-bis(1-piperazinylcarbonyl)-benzoic acid
Carboxylic acid group (end group)	2.66 2.92	5-[(phenylamino)carbonyl]-1,3-benzenedicarboxylic acid	2.81 2.91	5-(1-piperazynilcarbonyl)-1, 3-benzenedicarboxylic acid
Amino (end group)	4.23	*N*-(3-aminophenyl)-benzamide	8.48	1-benzoyl-piperazine

Because the IEP can be determined experimentally (*i.e.*, by zeta potential measurements), the actual challenge consists of finding an arrangement for each membrane containing 14 constituting units which takes into consideration the condition of “available” bonds, and has a certain distribution of charged groups which produce a theoretical IEP value equal or at least very similar to the experimental one.

**Figure 3:**
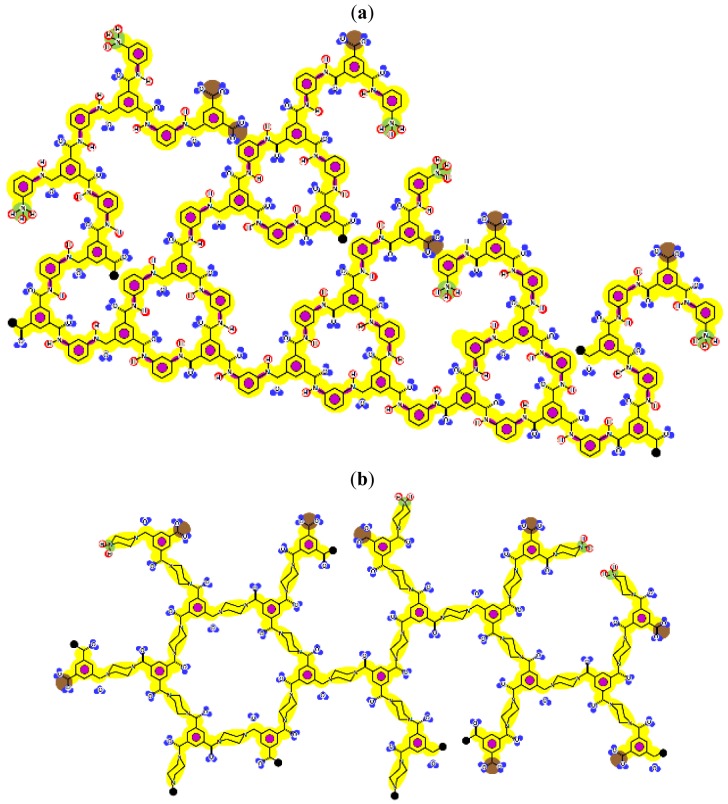
Pattern developed for **(a)** NF-90 membrane and **(b)** NF-270 membrane. Black points represent “available” bonds and the color code is the same as that of Figure 1.

Given that at least one terminal group (carboxylic acid or amine) should be present and the number of carboxylic acid groups should be at least equal to the number of linear constituting units, some iterative calculations were conducted and the patterns obtained are represented for both membranes in Figure 3. In each case, the existence of openings derived from the combination of linear and cross-linked constituting units was evidenced.

**Figure 4:**
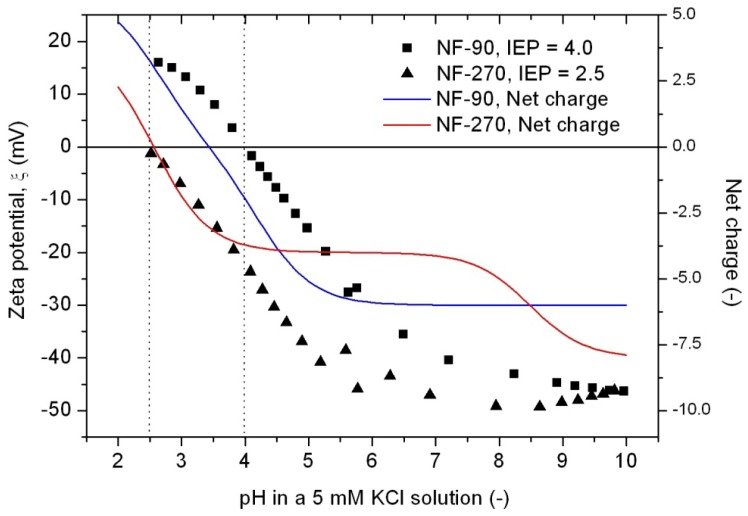
Zeta potential data and theoretical net charge for both NF-membranes.

Furthermore, IEP values equal to pH = 3.43 and pH = 2.56 were obtained for the NF-90 and the NF-270 membranes, respectively. These values are in good agreement with the experimental ones, especially those found for the NF-270 membrane (Figure 4). However, if the net charge is calculated for different pH values, very different tendencies are obtained to those which would be expected.

The curves exhibit a similar tendency around the IEP, but exhibit completely different behaviors at higher pH values. These tendencies suggest that each charged group definitively possesses its own pKa value, determined by the influence of the surrounding chemical structure but being actually impossible to determine in an reliable way. However, they can also indicate that charged groups in the monolayer underneath the surface monolayer also have an influence in the determination of both theoretical and experimental IEP values.

Due to the aim of this work being to propose a better picture for both nanofiltration membranes rather than to discover their real structures, both patterns represented in Figure 3 were considered for further study.

### 3.3. Understanding Membrane Characteristics: A New Approach Using T-SAR

**Figure 5:**
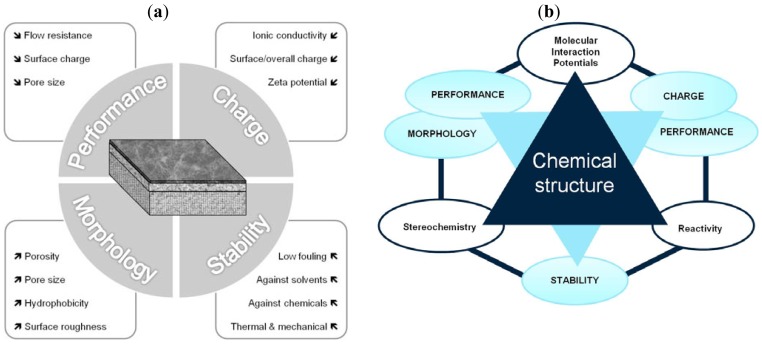
**(a)** Most important characteristics of nanofiltration membranes; and **(b)** A new approach using T-SAR to understand membrane characteristics.

Up to now, the traditional approach to characterize membranes focuses on the determination of four groups of parameters by using well-established methods, in order to explain membrane properties or the performance obtained in a specific application. These groups of parameters are: morphology, charge, performance and stability (Figure 5a). However, by following this traditional approach it is only possible to establish few and dispersed connections between chemical structure, properties and performance.

Based on the T-SAR analysis a new approach was developed to understand membrane characteristics from its chemical structure: The T-SAR triangle is combined with a second triangle comprising the four groups of parameters used for membrane characterization (Figure 5b). Once the T-SAR algorithm is successfully applied, a detailed analysis of membrane characteristics can be conducted, either by direct determination of such parameters or simply by using data already published for such membranes. As can be observed in Figure 5b, the data derived from at least two corners of the T-SAR triangle is required to explain each group of membrane characteristics:

- By combining stereochemistry data and the corresponding molecular interaction potentials, both morphology and performance (permeability) parameters can be discussed.
- The analysis of charge and performance (retention) parameters requires molecular interaction potentials together with reactivity data.
- The information related to stereochemistry and reactivity gives evidence to support the stability behavior of the membrane against thermal, mechanical and chemical agents.

In order to illustrate the use of the new approach and aiming to ascertain its advantages in comparison to the classical one, it will be employed to explain issues related to the roughness, hydrophobicity and pure water permeability of both FilmTec membranes.

The surface roughness can be derived from Atomic Force Microscopy (AFM) measurements, in which a three-dimensional picture of the membrane surface can be obtained. Values already published indicate that the surface roughness for the NF-90 membrane is manifestly higher that the surface roughness for the NF-270 membrane [27,28,29]. These results can be explained as a consequence of the cross-linking degree of monolayers constituting the membrane surface.

By comparison of the chemical structures of both membranes, it can be observed that the NF-90 membrane pattern (Figure 3a) contains only five available bonds, which allow certain flexibility on the resulting surface. Then, those constituting units near the available bonds constitute deep regions in the surface (they are connected with the underneath monolayer), while the units which are not suffering the strain derived from the available bonds, are oriented outside the surface. This behavior is in agreement with the typical ridge-and-valley structure observed by SEM for those membranes derived from trimesoyl chloride and aromatic diamines [30]. In contrast, the NF-270 membrane pattern (Figure 3b) contains more available bonds (eight) by the same number of constituting units, which tend to strain the structure and thus, to smooth down the resulting surface.

From AFM measurements [27] and also by using retention data of uncharged solutes [31], it is possible to estimate the pore size of membranes. From both references it is known that the pore size for the NF-270 membrane is slightly higher than for the NF-90 membrane, with all values ranging from 0.30 to 0.70 nm. However, it is known that pore sizes derived from AFM measurements can be underestimated due to the fact that the tip cannot probe into the depth of the pore, while pore sizes derived from retention data are subject to the limitations of the available models to describe separation performance [32].

As mentioned above, the existence of openings in both membrane patterns was confirmed (Figure 3) and they are represented in detail in Table 7. Such molecular structures were drawn using ISIS™/Draw 2.4 (MDL Information Systems, Inc.) with bond outside the opening represented as a carboxylic acid end-group. Then, these structures were optimized using HyperChem™ 7.5 (Hypercube, Inc.) or MOPAC2009™ (Stewart Computational Chemistry). Finally, optimized structures were processed with JMol 11.8.7 (an open-source Java 3D-viewer for chemical structures).

**Table 7:** Nano-openings resulting from the chemical structure of nanofiltration membranes.

Opening chemical structure	NF-90	NF-270
Considering the molecular interaction potentials according to the T-SAR color code	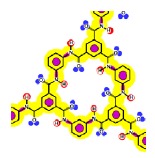	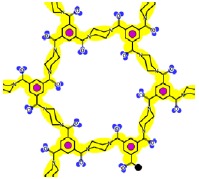
Considering an optimized chemical structure by using HyperChem™ 7.5 or MOPAC2009™	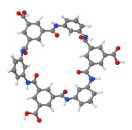	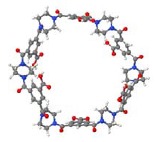
Considering the Van der Waals surface area determined by using JMol 11.8.7	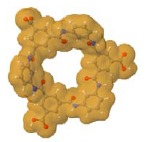	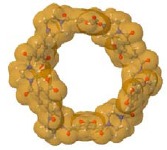

The opening observed in the structure of the NF-270 membranes was already described in the literature for polypiperazine amides, but no details about how such a structure was established were given [33]. No previous references were found for the opening observed in the structure of the NF-90 membrane. Finally, by measuring the distance between two opposite atoms and considering the existence of the Van der Waals surface area, the following opening sizes could be estimated: 0.80 nm for the NF-90 membrane and 1.30 nm for the NF-270 membrane. According to the aforementioned picture that the top layer is formed by several monolayers and also considering that every monolayer contains such openings, then the existence of nanopores in these kinds of membranes (responsible for the sieving effect) can be thus explained as the connection between nano-openings located in adjacent monolayers. However, such polymeric monolayers can be deformed under pressure and/or the presence of solvent. Consequently, these pores are dynamic in time and space, and should be conceived as free volume elements rather than permanent pores [34].

Hydrophobicity can be also described using the T-SAR approach. Theoretically, and according to the identified molecular interaction potentials for each membrane (Figure 3, Table 7), the NF-90 membrane exhibits both H-donor and H-acceptor potentials, the same potentials exhibited by the water molecule. That means that the NF-90 membrane must interact easily with water and the swelling process should occur rapidly. In contrast, interaction between the NF-270 membrane and water molecules are less favorable because H-donor potentials are absent in its structure, leaving more parts of the chemical structure which exhibit hydrophobic interaction potential in contact with water. In this case, a slower swelling process is expected and it could be evidenced during the determination of the zeta potential for the NF-270 membrane. It was found that the zeta potential varied 30% and 70% after 90 minutes and 3 days exposure to aqueous solution respectively, when compared to the zeta potential measured after instantaneous exposure to aqueous solution. Such phenomena were not observed for the NF-90 membrane.

As a consequence of this analysis and considering that the contact angle is a measure of the wettability of the membrane, it was found that the contact angle measurements carried out using the captive bubble method (using wet membranes) produce results which are in agreement with the T-SAR expected results. Consequently, the NF-270 membrane exhibits a higher hydrophobicity than the NF-90 membrane [35,36]. In contrast, the contact angle values determined by the sessile drop method (typically using dry membranes) are in clear contradiction which the expected results from chemical structure [24,28,37].

**Figure 6:**
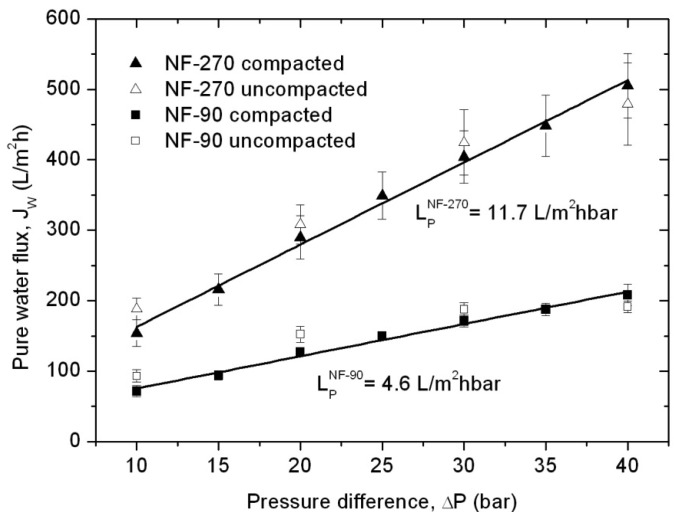
Pure water permeability determination. Full dots represent values for compacted membranes, while open dots are values obtained during membrane compaction.

Finally, the combination of membrane morphology and molecular interaction potentials already described can help to explain the differences in pure water permeability, which is the simplest performance parameter for such membranes. In Figure 6, water flux values for uncompacted and compacted membranes are represented as a function of the transmembrane pressure difference.

Water fluxes obtained during the compaction process are slightly higher than those obtained for the same membrane after compaction, except for those water fluxes obtained at the highest pressure difference used, which are slightly lower and indicate the status of compaction. Linear regressions for the compacted membrane values provide values for the pure water permeabilities, resulting in a higher value for the NF-270 membrane (11.7 L/m^2^hbar) compared with the NF-90 membrane (4.6 L/m^2^hbar).

These results are in qualitative agreement with our previous analysis. On the one hand, the NF-90 membrane possesses smaller pores and higher roughness than the NF-270 membrane. Additionally, the NF-90 top layer is thicker than the NF-270 top layer [38], a factor which is also contributing to the reduction in the water permeability. On the other hand, the NF-90 membrane exhibits stronger interactions with the water molecules due to the formation of hydrogen bonds, while these interactions are weaker for the NF-270 membrane. All of these effects complicate the flow of water through the membrane, leading to a smaller water flux for the NF-90 membranes at a given pressure difference.

### 3.4. Predicting Membrane Performance: The Case of Ionic Liquids

As a final discussion subject, the developed picture for nanofiltration membranes will be used to understand the separation of ionic liquids. In general, membranes derived from aromatic diamines (NF-90) show lower water fluxes, but higher retentions than those derived from aliphatic diamines (NF-270) [39]. This could be explained mainly due to the size of the nano-openings already discussed.

Several studies have demonstrated that a biphasic ionic liquid/water system can improve the asymmetric reduction of prochiral ketones, either using isolated enzymes [40,41] or whole cells [42,43,44,45,46]. However, to the best of our knowledge, there are no earlier references about the use of nanofiltration for the separation of hydrophobic ionic liquids from aqueous solution. Consequently, three ionic liquids containing the (CF3SO2)2N anion were selected due to their performance during the biotransformation of 2-octanone into (R)-2-octanol in a 200 mL reaction system. They exhibited higher conversion (91% for IM16, 86% for Pyr16 and 78% for Py6) compared with the traditional one-phase system (55% for aqueous buffer) after five hours of reaction (unpublished results).

Then, the first aspect to be considered is the size of the ionic liquids. Ionic volumes were determined by using BP86/TZVP + COSMO calculations [47], while the ionic radius was calculated from the ionic volume assuming ideally spherical behavior [48], as follows:

(3) $${\text{r}}_{\text{ION}}^{\pm }=\sqrt[{3}]{\frac{3\cdot {\text{V}}_{\text{ION}}^{\pm }}{4\pi }}$$

where:

r^±^_ION_: ionic radius (nm);
V^±^_ION_: ionic volume (nm^3^).

Both ionic volume and ionic radius for the ionic liquids considered in this study are summarized in Table 8, ordered by increasing size. The anion used is almost as big as the cations used, which exhibit a similar size despite being derived from different head-groups (pyridinium, imidazolium and pyrrolidinium).

**Table 8:** Ionic volumes and radii for selected cations and anions.

**Ionic liquid entity**		**Ionic volume, V^±^_ION_ (nm^3^)**	**Ionic radius, r^±^_ION_ (nm)**
Anion	(CF3SO2)2N	0.2178	0.373
Cations	Py6	0.2387	0.385
	IM16	0.2438	0.388
	Pyr16	0.2589	0.395

In this context, the molecular diameter for an ionic liquid can be calculated as the sum of anionic and cationic diameters [48], as follows:

(4) $${\text{d}}_{\text{m}}=2\cdot {\text{r}}_{\text{m}}=2\cdot {\text{r}}_{\text{ION}}^{-}+2\cdot {\text{r}}_{\text{ION}}^{+}$$

where:

d_m_: molecular diameter (nm);
r_m_: molecular radius (nm);
r^−^_ION_: anionic radius (nm);
r^+^_ION_: cationic radius (nm).

Because the molecular diameter does not consider the hydration of the ionic liquid, the calculated values summarized in Table 9 can be compared with the sizes of the membrane openings when the Van der Waals surface is considered (0.80 nm for NF-90 and 1.30 nm for NF-270).

**Table 9:** Molecular diameter and interaction potentials for three different hydrophobic ionic liquids.

**Ionic liquid**	**Molecular diameter, dm (nm)**	**Cation**	**Anion**
Py6 (CF3SO2)2N	1.516	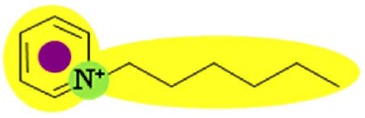	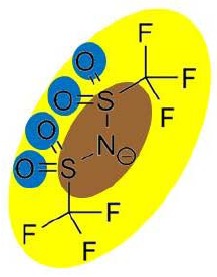
IM16 (CF3SO2)2N	1.522	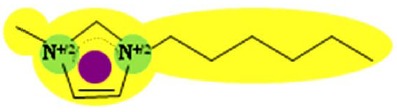	
Pyr16 (CF3SO2)2N	1.536	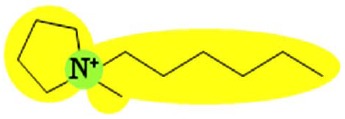	

According to this, most of the ions constituting these hydrophobic ionic liquids should be retained by the NF-90 membrane due to their bigger size compared with that of the membrane opening. However, in the case of the NF-270 membrane, it has to be taken into account that the molecular diameter consideration assumes that anion and cation remain together due to charge interactions, but their permeation through the openings may not necessarily occur simultaneously.

In order to explain the differences in retention by the NF-270 membrane, molecular interaction potentials of the hydrophobic ionic liquids were also represented in Table 9. They are quite different, due to the differences in the cation structures, while the interaction potentials associated to the (CF3SO2)2N anion are a hydrophobic potential at the fluorinated carbon atoms, a delocalized negative charge due to resonance effects and an H-acceptor potential at the oxygen atoms.

In the case of the NF-270 membrane, repulsions occur between the (CF3SO2)2N anion and the membrane due to both negative charge (brown-colored) and H-bonding acceptors (blue-colored) in both chemical structures. Then, these repulsions together with the openings size lead mainly to high retentions for such ionic liquids, but the influence of the cation employed should be also considered.

Both Py6 and IM16 cations exhibit hydrophobic potential and charge transfer potentials, but with an important difference: in the case of Py6 the positive charge is localized at the nitrogen atom, while in the case of IM16 there is a delocalized positive charge between both nitrogen atoms. The Pyr16 cation exhibits hydrophobic potential but not charge transfer due to its aliphatic ring, with a localized positive charge which can be partially sheltered because both methyl and hexyl side chains are bound to the same nitrogen atom. From logk_0_ values derived from reversed phase gradient HPLC retention times and used as an approximate measure of cation lipophilicity, the following values are known: 1.04 for Py6 (unpublished data), 1.24 for IM16 and 1.17 for Pyr16 [49].

Pyr16 cation exhibits interactions mainly due to the hydrophobic attractions with the NF-270 membrane structure. Both IM16 and Py6 cations experience also charge transfer, which is not present in the Pyr16 cation. Furthermore, the charge delocalization in the IM16 cation leads to weaker attractions with the negatively charged groups of the membrane, in comparison with the other two cations possessing localized positive charge.

Considering that T-SAR is a tool for qualitative prediction, the following tendencies can be summarized in order of importance:

- Cation size: Pyr16 > IM16 > Py6;
- Hydrophobicity: IM16 ≥ Pyr16 > Py6;
- Charge transfer potential: Py6 > IM16;
- Cation positive charge: Py6 > Pyr16 ≥ IM16.

With cation size and hydrophobicity playing the most important roles in the separation, the retention should decrease as follows: Pyr16 > IM16 > Py6. Finally, the higher the retention (due to less interactions with the membrane structure), the higher the permeate flux should be. Consequently, the tendency Pyr16 > IM16 > Py6 is also valid for permeate fluxes.

In order to verify the reliability of these predictions, some filtration tests were carried out with the NF-270 membrane (Table 10). The values obtained for Py6 (CF3SO2)2N reflect also the influence of the concentration employed (almost twice the feed concentration used for Pyr16 and IM16 cations). Due to the presence of more ions both retention and permeate flux are considerably lower than those expected for the same concentration level. However, the experimental values obtained for the filtration of such ionic liquids are in good agreement with the predictions summarized above.

**Table 10:** Experimental data related to the filtration of hydrophobic ionic liquids with the NF-270 membrane (80% recovery rate, 35 bar, IL-saturated feed solutions).

	**Ionic liquid**		
	
**Parameter**	**Pyr16** **(CF3SO2)2N**	**IM16** **(CF3SO2)2N**	**Py6** **(CF3SO2)2N**
Retention (%)	97.4	94.7	88.2
Permeate flux (L/m^2^h)	220	117	88

## 4. Conclusions

Based on the novel methodology to understand the chemical functionality of active membrane layers that derived from the T-SAR approach, it can be now qualitatively predicted both related properties and separation performance for different solute classes, not only ionic liquids.

At first sight, this qualitative analysis provides a tangible picture of a nanofiltration membrane which shakes off the black-box concept so far used for such membranes. Admittedly, this new picture is only a small contribution possessing its own limitations. However, it can be assumed to provide a new avenue to systematically design task-specific nanofiltration membranes.

Additionally, and mainly due to its simplicity, the proposed picture could also serve as a meeting point for engineers, chemists, scientists and technicians working in the field of membrane technology. It could also encourage further developments through interdisciplinary work, especially in the fields of membrane synthesis and membrane characterization.
